# How does social support shape the association between depressive symptoms and labour market participation: a four-way decomposition

**DOI:** 10.1093/eurpub/ckab185

**Published:** 2021-12-06

**Authors:** Karin Veldman, Ronnie Pingel, Johan Hallqvist, Christopher G. Bean, Anne Hammarström

**Affiliations:** 1 Department of Health Sciences, Community & Occupational Medicine, University of Groningen, University Medical Center Groningen, Groningen, The Netherlands; 2 Department of Statistics, Uppsala University, Uppsala, Sweden; 3 Department of Public Health and Caring Sciences, Uppsala University, Uppsala, Sweden; 4 School of Psychology, University of Adelaide, Australia; 5 Institute of Environmental Medicine, Karolinska Institutet, Stockholm, Sweden; 6 Department of Epidemiology and Global Health, Umeå University, Umeå, Sweden

## Abstract

**Background:**

Little is known about factors that may explain the association between depressive symptoms and poor labour market participation (LMP). The aim of this study is to examine the mediation and interaction effects of social support on the association between depressive symptoms and LMP.

**Methods:**

Data were used from 985 participants (91% of the initial cohort) of the Northern Swedish Cohort, a longitudinal study of Swedish participants followed from adolescence throughout adulthood. Depressive symptoms were measured at age 16, social support at age 21 and LMP from age 30 to 43. Poor LMP was defined as being unemployed for a total of 6 months or more between the ages of 30 and 43. A four-way decomposition approach was applied to identify direct, mediation and interaction effects, together and separately.

**Results:**

Both depressive symptoms during adolescence and social support at young adulthood were associated with poor LMP [odds ratio (OR) = 1.70, 95% confidence interval (CI) 1.17–2.47 and OR = 2.56, 95% CI 1.78-3.68 respectively]. The association between depressive symptoms and poor LMP was partially mediated by a lack of social support. No interaction effect of a lack of social support was found.

**Conclusion:**

The results suggest that depressive symptoms influence not only later LMP but also the intermediary level of social support, and in turn influencing later LMP. Recommendations for public health are to detect and treat depressive symptoms at an early stage and to focus on the development of social skills, facilitating the increased availability of social support, thereby improving future LMP.

## Introduction

Several studies have shown that depressive symptoms increase the risk of adverse labour market outcomes.^1–7^ However, it is unclear which factors may influence the association between depressive symptoms and labour market participation, and whether these factors play a mediating (i.e. indirect) or moderating (i.e. interaction) role.

Bronfenbrenner’s bioecological theory of human development may be helpful to improve our understanding of the impact of early depressive symptoms on poor labour market participation in adulthood.^8^ Therefore, the present study applies the PPCT model (Process, Person, Context and Time) to examine the developmental effects of personal and environmental characteristics (i.e. the context), while also taking into account development over the life course (i.e. time).

Consideration of personal characteristics is of great importance for understanding human development. In this study, depressive symptoms and employment status are seen as personal characteristics. A number of studies have shown that individuals with depressive symptoms are at risk of adverse labour market outcomes, including unemployment.^1–7^ It is suggested that individuals with depressive symptoms may enter a downward spiral, starting with lower educational levels^9–13^ and subsequently with worse employment conditions (e.g. higher unemployment rates and lower income). Individuals experiencing depressive symptoms are at risk of recurrent episodes of depression,^14^^,^^15^ and so it is important to broaden our knowledge about the influence of social processes on these personal characteristics.

According to Bronfenbrenner, human development can be better understood when taking into account the context of the individual. The course and consequences of depressive symptoms are affected by the context wherein an individual operates.^16^^,^^17^ Previous research suggests that depressed individuals experience better outcomes when they feel appreciated by their family and peers, implying that contextual factors have a great influence on the course of depressive symptoms.^16^^,^^17^ A lack of social support may therefore be a risk factor that is both related to depressive symptoms and poor labour market participation. Social support has been described as ‘the experience of being cared for, loved, esteemed, and valued by others and being part of a social network’.^18^ Examining the effect of both depressive symptoms and social support holds promise for reducing the harmful impact of early depressive symptoms on poor labour market participation later in life. However, little is known as to how social support may shape the association between depressive symptoms and poor labour market participation. In Bronfenbrenner’s theory, processes are seen as ‘the primary engines of development’ and refer to the interplay between a person and their context.^8^ Several studies have shown that people experiencing depressive symptoms are more likely to report disruptive interpersonal relationships, perceive less social support and lack social skills.^17^^,^^19–21^ Although evidence for poor social support as a risk factor for unemployment is lacking, several studies have found an association between poor social skills and poor labour market participation. Good social skills have been shown to be of major importance for the acquisition and maintenance of employment^22–26^ and are also likely to be a prerequisite for access to effective social support.^17^^,^^27^ Furthermore, social support could be related to poor labour market participation as social networks play an important role for finding a job.^28^^,^^29^ It is therefore likely that depressive symptoms and a lack of social support may both act as risk factors for poor labour market participation. As such, we hypothesize that a lack of social support will reinforce the effect of depressive symptoms on adult labour market participation. In other words, individuals with depressive symptoms who experience good social support will have a lower risk of poor labour market participation, compared with those with depressive symptoms and a lack of social support (i.e. a moderating effect).

Time is another aspect of the PPCT model, which refers not only to chronological process but also to the ordering of life events.^30^ In Bronfenbrenner’s theory, time is employed to ‘identify the impact of prior life events and experiences, singly or sequentially, on subsequent development’ (p. 83). This life course perspective allows us to examine the effect of subsequent harmful exposures during the life course. Since previous research suggests that people who experience depressive symptoms also experience lower social support and feel more isolated,^17^^,^^19–21^ it is hypothesized that poor social support will mediate the association between depressive symptoms and employment status. In other words, it is expected that depressive symptoms in adolescence will have not only a direct effect on the risk of later poor labour market participation but also an indirect effect whereby those with depressive symptoms will experience a lack of social support, and this lack of social support will increase the risk of poor labour market participation (i.e. a mediating effect).

From previous research, it is known that depressive symptoms and social support are interrelated and both depressive symptoms and social support are associated with labour market participation.^17^^,^^19–23^^,^^25^ Therefore, besides a separate mediation and moderation effect, a third hypothesis is that there is a combined effect of depressive symptoms and social support on labour market participation (i.e. a mediated interaction). In the past decades, the development of innovative methods for mediation allows us to better understand underlying mechanisms for existing relationships.^31^ VanderWeele’s^32^ four-way decomposition approach facilitates the examination of both the mediation and interaction effects of social support, on the association between depressive symptoms and poor labour market participation, both separately and together. A better understanding of the interplay between depressive symptoms and social support and its combined effect on labour market participation may provide new insights for the development of targeted interventions. In addition, the distinction between mediation and interaction (or moderation) is of utmost importance for choosing the correct intervention policy, as mediation and interaction reflect two very different causal processes.^33^

The aim of this study is to examine how social support in young adulthood shapes the previously identified association between depressive symptoms in adolescence and poor labour market participation in adulthood. Beyond the direct effect of depressive symptoms on later poor labour market participation, this study considers an intermediary role of social support and evaluates whether the effect of social support on the association between depressive symptoms and poor labour market participation is due to interaction, a combination of interaction and mediation or mediation without interaction. By considering the different potential pathways, the four-way decomposition method provides proportionalities for these various effects, offering novel insights into the mechanistic relations between these variables, improving our ability to evaluate their public health importance.

## Methods

### Study design and sample

Data were sourced from the Northern Swedish Cohort (NoSCo), a longitudinal study of Swedish participants followed from adolescence and throughout adulthood. In 1981, participants were recruited during their final year of compulsory schooling in Luleå, a middle-sized municipality in Northern Sweden. In total, 1083 participants were eligible, of whom 1080 (99.7%) participated at baseline. With the baseline measurement in 1981, NoSCo has 27 years of follow-up, with subsequent measurement waves conducted at ages 18 (1983), 21 (1986), 30 (1995) and 43 (2008). Participants provided informed consent by completing the questionnaires. The Regional Research Ethics Review Board at Umeå University, Sweden, approved all NoSCo study protocols. Retention rates were high during follow-up: in 2008, 93.7% of the initial cohort participated at the latest measurement wave (*N* = 1010). More detailed information about the design, the sample and the procedures of NoSCo can be found elsewhere.^34^ The measures used in the present study are based on questionnaire data, obtained at age 16, age 21 and age 43.

### Measures

#### Outcome variable

*Labour market participation* from age 30 to age 43 years was measured by asking the participants about their labour market status between 1995 and 2007. For each half year, participants could mark one or more options about their labour market status. The total number of options reported by the participants was divided by 6 (i.e. the number of months per half year). Next, for the total period of 12 years, all months of no employment (i.e. unemployment, active labour market policy measure, outside labour market) were summed. Participants were divided into two groups: those with poor labour market participation for less than 6 months (=0) or poor labour market participation for 6 months or more (=1).

#### Exposure variable

*Depressive symptoms* were measured at age 16 years with six questions about: sleeping problems, poor appetite, general tiredness, feeling down and sad, dejected about the future and concentration problems (α = 0.73, inter-item correlation ranging from 0.26 to 0.51). Participants rated their level of depressive symptoms on a four-point scale (0 = never, 3 = always). The middle response options were combined. The mean value of the six items was calculated, and higher scores indicate a higher level of depressive symptoms. The variable was dichotomized at the 80th percentile into 0 (=no severe depressive symptoms) and 1 (=severe depressive symptoms).

#### Mediating and moderating variable

*Lack of social support* was measured at age 21 years by asking the participants whether they felt appreciated by the people around them or not. The following five questions were asked: ‘Generally speaking, are other people nice to you?’, ‘Generally speaking, do other people appreciate what you do?’, ‘Do you usually get the feeling that people think your presence is unnecessary?’, ‘Do you think it is hard for other people to listen to what you are saying?’, ‘Generally speaking, do other people understand or respect you?’. Response options were (i) yes, very often, (ii) yes, quite often, (iii) no, occasionally, (iv) no, hardly ever. An index score was calculated by summing the items and subsequently dividing the sum score by the number of items (α = 0.80, inter-item correlation ranging from 0.32 to 0.50). Higher scores indicate a lower level of social support. The variable was dichotomized at the 90th percentile into 0 (=no lack of social support) and 1 (=lack of social support).

#### Confounding variables

*Sex* was retrieved from the school class records.

*Parental occupational group* was measured at age 16 and coded into two social groups, i.e. low (both parents manual workers) and medium/high (one or both parent(s) non-manual workers).

*Employment status* was measured at age 21 by asking the participants about their labour market status. Participants were divided into two groups: those who were not unemployed (=0) or those who were unemployed (=1).

### Data analyses

Descriptive statistics are presented for all variables for the whole sample and stratified by the severity of depressive symptoms and degree of social support. Differences between the exposure categories were assessed by Chi-square tests for categorical variables (i.e. sex, parental occupational class, employment status at age 21 and depressive symptoms) and F-tests in one-way analysis of variance analyses for the continuous variable (i.e. social support).

Second, with logistic and linear regression analysis, the univariate and multivariate associations between depressive symptoms, social support and unemployment were examined.

Third, four-way decomposition was applied to examine the association between depressive symptoms and unemployment and the potential influence of a lack of social support.^32^ The total excess relative risk (ERR) was decomposed into four components:

1. The effect of depressive symptoms on unemployment, while controlling for the level of social support, i.e. the Controlled Direct Effect (CDE).
2. The combined effect of depressive symptoms and a lack of social support on unemployment, that is attributable to interaction but not mediation, i.e. the Reference Interaction (INT_ref_).
3. The combined effect of depressive symptoms and lack of social support on unemployment, that is attributable to both interaction and mediation, i.e. the Mediated Interaction (INT_med_).
4. The effect of depressive symptoms on unemployment that is purely mediated by a lack of social support, i.e. the Pure Indirect Effect (PIE).

For the four-way decomposition, a Poisson regression model was used, and only participants with complete data for all items were included in the analyses (*N* = 985). Model 1 represents the crude model, and Model 2 was adjusted for sex, parental occupational class and employment status at age 21. All confounding variables are likely to be associated with the outcome, the exposure, and the mediating and moderating variable and were included in the analyses. Data on parental occupational group were missing for five participants, and these participants were excluded from the analysis. A sensitivity analysis was performed with social support as a continuous variable. Analyses were conducted using SPSS version 25.0 and Stata version 15.^35^

## Results

### Sample characteristics

The total sample consisted of 989 participants (51.8% men). Almost 30% of the participants’ parents had low occupational class. Participants who experienced severe depressive symptoms were more often unemployed for 6 months or more (34.1% vs. 21.4%, *P* ≤ 0.001), were more likely to report a lack of social support (29.1% vs. 14.7%, *P* ≤ 0.001), compared with participants who experienced no severe depressive symptoms. Table  1 provides the background characteristics for the total sample and by the severity of depressive symptoms and lack of social support.

**Table 1:** Descriptive information of the background and exposure and moderating variables for the total sample and by employment status (*N* = 985)

		Exposure categories				
	Total	ab^a^	Ab^a^	aB^a^	AB^a^	*P*-value
	*N* (%)	*N* (%)	*N* (%)	*N* (%)	*N* (%)	
Total sample		699	121	117		
Background variables						
Sex						<0.001
Men	513 (52.1)	386 (55.2)	42 (34.7)	65 (55.6)	20 (41.7)	
Women	472 (47.9)	313 (44.8)	79 (65.3)	52 (44.4)	28 (58.3)	
Parental occupational groups						0.66
Low	299 (30.4)	218 (31.2)	35 (28.9)	35 (29.9)	11 (22.9)	
Medium/high	686 (69.6)	481 (68.8)	86 (71.1)	82 (70.1)	37 (77.1)	
Employment status (age 21)						<0.001
Unemployed	855 (86.8)	79 (11.3)	13 (10.7)	29 (24.8)	9 (18.8)	
Not unemployed	130 (13.2)	620 (88.7)	108 (89.3)	88 (75.2)	39 (81.2)	
Outcome variable						
Labour market participation						<0.001
Successful	767 (76.0)	569 (81.4)	86 (71.1)	72 (61.5)	24 (50.0)	
Poor	238 (24.0)	130 (18.6)	35 (28.9)	45 (38.5)	24 (50.0)	

a ab: No severe depressive symptoms and no lack of social support | Ab: Severe depressive symptoms and no lack of social support | aB: No severe depressive symptoms and lack of social support | AB: Severe depressive symptoms and lack of social support.

### Associations among depressive symptoms, social support and employment status

The results (see table 2) show that both depressive symptoms during adolescence and social support at young adulthood were associated with adult unemployment [odds ratio (OR) = 1.97, 95% confidence interval (CI) 1.37–2.81 and OR = 2.85, 95% CI 2.00–4.06–3.68, respectively). Depressive symptoms at adolescence were also associated with lack of social support in young adulthood (OR = 2.44, 95% CI 1.64–3.63). Adjustment for sex, parental occupational class and employment status at age 21 did not have a notable influence on these results.

**Table 2.** Logistic regression analysis of depressive symptoms, social support and labour market participation

	Labour market participation				Social support			
	Model 1^a^		Model 2^b^		Model 1^a^		Model 2^b^	
	OR	95%CI	OR	95%CI	OR	95%CI	OR	95%CI
Depressive symptoms								
No severe	1		1		1		1	
Severe	1.97	1.37–2.81	1.70	1.17–2.47	2.37	1.61–3.49	2.44	1.64–3.63
Social support	2.85	2.00–4.06	2.56	1.78-3.68				

a Crude model.

b Model 1 + adjustment for sex, parental occupational groups and employment status at age 21.

### A four-way decomposition of the association between depressive symptoms, lack of social support and unemployment

The decomposition of the association between depressive symptoms, social support and unemployment is presented in table 3. It shows that there was an effect of depressive symptoms on poor labour market participation [total effect: ERR = 0.61, 95% CI 0.21–1.01).

**Table 3.** Total and decomposed effects of depressive symptoms on poor labour market participation due to mediation and/or interaction with social support

	Model 1^a^						Model 2^b^					
	TERR	95%CI	Component	ERR	95%CI	Proportion Attributable	TERR	95%CI	Component	ERR	95%CI	Proportion Attributable
Depressive symptoms												
No severe	1						1					
Severe	1.61	1.22 to 2.01					1.58	1.18 to 1.98				
			CDE	0.52	0.11 to 0.92	76.6%			CDE	0.49	0.08 to 0.90	75.7%
			INT_ref_	0.01	−0.12 to 0.13	1.3%			INT_ref_	0.006	−0.19 to 0.08	1.2%
			INT_med_	0.01	−0.11 to 0.13	1.3%			INT_med_	0.004	−0.08 to 0.15	1.2%
			PIE	0.11	0.04 to 0.19	20.8%			PIE	0.11	0.04 to 0.19	21.9%
			TE	0.61	0.21 to 1.01				TE	0.49	0.18 to 0.98	

TERR, total effect risk ratio; ERR, excess relative risk; CDE, controlled direct effect; INT_ref_, reference interaction; INT_med_, mediated interaction; PIE, pure indirect effect; TE, total effect.

a Crude model.

b Model 1 + adjustment for sex, parental occupational groups and employment status at age 21.

These results indicate that the direct effect of depressive symptoms in adolescence, after controlling for a lack of social support, was an increased risk of poor labour market participation in adulthood (CDE: ERR = 0.52, 95% CI 0.11–0.92). A lack of social support in young adulthood mediated the association between depressive symptoms and poor labour market participation suggesting that depressive symptoms lower the level of social support, and a lack of social support increases the risk of poor labour market participation (PIE: ERR = 0.11, 95% CI 0.04–0.19). The overall proportion attributable to mediation is 20.8% (the sum of the pure indirect effect and mediated interaction, divided by the total effect). A lack of social support did not moderate the association between depressive symptoms and poor labour market participation. Adjustment for sex, parental occupational class and employment status at age 21 did not have a notable influence on these results (table 3).

### Sensitivity analysis

The results of the sensitivity analysis were consistent with those presented, but the latter provide greater interpretability (data are available on request).

## Discussion

Depressive symptoms in adolescence were associated with poor labour market participation in adulthood, and a lack of social support in young adulthood mediated the association between depressive symptoms and poor labour market participation. These findings provide support for the emphasis Bronfenbrenner’s model places on the importance of proximal processes (i.e. the interplay between a person and the immediate context) in bioecological theory.^8^ Furthermore, the time component of his model was also shown to be important, as adolescent depressive symptoms precede a lack of social support in young adulthood and consequently increase the risk on poor labour market participation.

Our results regarding the association between depressive symptoms and poor labour market participation are consistent with previous research.^1^^,^^2^^,^^4^^,^^5^ Previous studies have tended to involve shorter periods of follow-up, and our results show the long-term consequences of depressive symptoms in adolescence. Furthermore, our results suggest that depressive symptoms influence not only later labour market participation but also the intermediary level of social support. Participants with depressive symptoms in adolescence reported lower levels of social support in young adulthood. These results suggest that having depressive symptoms makes it more difficult to acquire and access social support. The social interplay between an individual and the context may be hampered by the presence of depressive symptoms, as people with depressive symptoms may also lack social skills that are necessary for them to access social support.^16^^,^^17^^,^^20^

It is conceivable that individuals with early depressive symptoms may enter a downward spiral, affecting both their social relationships and personal characteristics, as poor labour market participation and recurrent depressive symptoms. Social skills are highly valued in the workplace, and prior research suggests that people with disabilities may lose their job mainly because of a lack of social skills, and not because they could not perform their tasks.^35^^–^^37^ Another explanation might be that people with depressive symptoms are at greater risk of discrimination in the labour market, where employers tend to prefer employees without depressive symptoms.^38^

We found no interaction effect of social support, suggesting that there is no additional increase in susceptibility for poor labour market participation among people who experience depressive symptoms in adolescence when combined with exposure to a lack of social support in young adulthood. It could also be that our sample may be underpowered to detect statistically significant interaction effects.

The present study has several strengths. We used data from a population sample with 27 years of follow-up. Retention rates were very high, with 94.3% of the original cohort who were still alive, participating at the 27-year follow-up. The results of this study have to be considered in the light of some limitations. Ideally, we would have examined whether the impact of social support varies between contexts. It might be that social support of peers is valued higher than social support of family members, or the other way around. Consideration of different elements of the microsystem is also consistent with Bronfenbrenner’s recommendations^39^; however, in our data, questions about social support were not contextualized, i.e. it is unknown whether respondents evaluated their relationships with specific reference to family members, friends, classmates or colleagues. Furthermore, the analyses were adjusted for sex and occupational class, but other factors, for example school grades, family status, may also have an influence on the examined associations. Due to issues related to statistical power, the number of confounding variables able to be included in the analyses was limited. Ideally, the analyses would have been adjusted for social support at age 16, but unfortunately, social support was not measured at age 16. This implies that the results should be interpreted with caution and underscores the importance of future research to examine this further.

The results of our study demonstrate the long-term consequences of depressive symptoms in adolescence, and the important role of social support. Research by Olesen et al.^2^ suggests that poor mental health is both a risk factor for and a consequence of poor labour market participation. It is also shown by Axelsson and Ejlertsson^40^ that unemployed people with low social support reported more mental health problems, compared with unemployed people with high social support. It is therefore important not only to detect and treat depressive symptoms at an early stage but also to focus on the development of social skills among those with depressive symptoms.

Applying the four-way decomposition approach of VanderWeele,^32^ we provide a new perspective on the understanding of the underlying mechanism between depressive symptoms and poor labour market participation. In the present study, depressive symptoms were found to be a risk factor for both a lack of social support and poor labour market participation. Moreover, the association between depressive symptoms and poor labour market participation was partly mediated by a lack of social support. These findings highlight the importance of further research to examine how social support shapes the association between depressive symptoms and poor labour market participation, as well as identifying a lack of social support as a potential target for intervention.
